# Bacterial profile of surgical site infection and antimicrobial resistance patterns in Ethiopia: a multicentre prospective cross-sectional study

**DOI:** 10.1186/s12941-023-00643-6

**Published:** 2023-11-07

**Authors:** Seble Worku, Tamrat Abebe, Ashenafi Alemu, Berhanu Seyoum, Göte Swedberg, Alemseged Abdissa, Adane Mihret, Getachew Tesfaye Beyene

**Affiliations:** 1 Department of Medical Laboratory Science, College of Medicine and Health Sciences, Debre Tabor University, Debre Tabor, Ethiopia; 2 Bacterial and Viral Diseases Research Directorate, Armauer Hansen Research Institute, Addis Ababa, Ethiopia; 3 Department of Microbiology, Immunology and Parasitology, College of Health Sciences, School of Medicine, Addis Ababa University, Addis Ababa, Ethiopia; 4 Department of Medical Biochemistry and Microbiology, Uppsala University, Uppsala, Sweden

**Keywords:** Surgical site infections etiologies, Emerging pathogens, Multidrug resistance, Risk factors, Ethiopia

## Abstract

**Background:**

Globally, surgical site infections (SSI) are the most commonly reported healthcare-associated infections.

**Methods:**

A multicentre study was conducted among patients who underwent surgical procedures at four hospitals located in Northern (Debre Tabor), Southern (Hawassa), Southwest (Jimma), and Central (Tikur Anbessa) parts of Ethiopia. A total of 752 patients clinically studied for surgical site infection were enrolled. The number of patients from Debre Tabor, Hawassa, Jimma, and Tikur Anbessa, hospitals was 172, 184, 193, and 203, respectively. At each study site, SSI discharge culture was performed from all patients, and positive cultures were characterized by colony characteristics, Gram stain, and conventional biochemical tests. Each bacterial species was confirmed using Matrix-Assisted Laser Desorption/Ionization Time-of-Flight Mass Spectrometry (MALDI TOF). An antimicrobial susceptibility test (AST) was done on Mueller–Hinton agar using the disk diffusion method. Logistic regression analysis was used to assess associations of dependent and independent variables. A p-value < 0.05 was considered statistically significant. Data were analysed using STATA 16 software.

**Results:**

Among 752 wound discharge cultures performed, 65.5% yielded growth. Among these, 57.9% and 42.1% were Gram-negative and Gram-positive isolates, respectively. In this study, a total of 494 bacteria were isolated; *Staphylococcus aureus* (31%), *Escherichia coli* (20.7%), and *Klebsiella pneumoniae* (9.8%) were the most common. Rare isolates (0.8% each) included *Raoultella ornithinolytica, Stenotrophomonas maltophilia*, *Alcalignes faecalis, Pantoea ecurina, Bacillus flexus, and Paenibacillus tylopili*. *Enterobacteriaceae* showed high levels of resistance to most of the tested antibiotics but lower levels of ertapenem (32.9%), amikacin (24.3%), imipenem (20.3%), and meropenem (17.6%) resistance. Multidrug-resistant (MDR) frequency of *Enterobacteriaceae* at Debre Tabor, Hawassa, Jimma, and Tikur Anbessa hospitals was 84.5%, 96.5%, 97.3%, and 94%, respectively. Ages ≥ 61 years (AOR = 2.83, 95% CI: 1.02–7.99; P 0.046), prolonged duration of hospital stay (AOR = 4.15, 95% CI: 2.87–6.01; P 0.000), history of previous antibiotics use (AOR = 2.83, 95% CI: 1.06–2.80; P 0.028), history of smoking (AOR = 2.35, 95% CI: 1.44–3.83; P 0.001), emergency surgery (AOR = 2.65, 95% CI: 1.92–3.66; P 0.000), and duration of operation (AOR = 0.27, 95% CI: 0.181–0.392; P 0.000) were significant risk factors.

**Conclusion:**

The most prevalent isolates from Gram-positive and Gram-negative bacteria across all hospitals were *S. aureus* and *E. coli,* respectively*.* Many newly emerging Gram-negative and Gram-positive bacteria were identified. Variation between hospitals was found for both SSI etiology type and MDR frequencies. Hence, to prevent the emergence and spread of MDR bacteria, standard bacteriological tests and their AST are indispensable for effective antimicrobial stewardship.

## Introduction

Surgical site infection (SSI) is the major costliest healthcare-associated infection and a substantial cause of morbidity and mortality throughout the world [1, 2]. It occurs near or at the incision site and/or deeper underlying tissue spaces and organs within 30 days of a surgical procedure performed (or up to 90 days for implanted prosthetics) [3]. In low and middle-income countries SSI ranked the most frequently reported case of nosocomial infections [4], and in some settings, up to one-third of patients who are operated on [5] can catch SSI, despite standard protocols of preoperative preparation and antibiotic prophylaxis are practiced [6]. The SSI rate in Ethiopia has been reported to be between 14.8 and 20% [5, 7–9], and surgical patients account for 38% of general surgical wards at various teaching hospitals [10]. It results from mostly bacterial contamination during or after the surgical procedure but only a small portion progresses to clinical infection due to innate host defences removing contaminants. The contamination that will lead to surgical site infection depends on the dose of bacterial contamination, the virulence, and drug resistance of the bacteria [11]. Most SSI infections are preventable [11], however probable development of an infection depends on the age, immunocompromising conditions of the host, or the antimicrobial-resistance (AMR) nature of the infecting microorganisms [12]. The frequency varies from one hospital to the other and is related to complications [13]. Patients with SSI are twice as likely to die, 60% more likely to spend time in an intensive care unit (ICU), and more than five times more likely to be readmitted to the hospital after discharge [14]. The most common pathogens associated with surgical wound infections are *Staphylococcus aureus, Escherichia coli,* Klebsiella spp., Proteus spp*.,* Citrobacter spp., Acinetobacter spp., Coagulase negative *Staphylococcus aureus* and *pseudomonas aeruginosa* [7, 15]. Beta-lactam antibiotics are the most widely used antibiotics for SSI prophylaxis and therapy; however, 30% to 90% of antibiotics are misused or overused [16, 17]. This inappropriate overuse increases selection pressure, favouring the emergence of drug-resistant bacteria, making the choice of empirical therapy more difficult and expensive, and poses a serious threat to public health, thus increasing the global risk of SSI [18, 19]. The condition is more serious due to irrational antimicrobial prescriptions and un-updated empirical therapy. Hence, the use of data from clinical laboratories' antibiotics susceptibility testing (AST) or solid epidemiological data from ongoing nosocomial infection surveillance is needed to minimize the problem [20]. In developing countries, including Ethiopia, published reports on bacterial pathogens and their antibiotics resistance patterns of frequently causing SSIs are relatively scarce [21] compared to the developed parts of the world. Besides, virtually all earlier reports depend on phenotypic laboratory methods to characterize pathogenic bacteria and studies were done at single sites with small sample sizes [9, 22, 23]. A recent systematic review and meta-analyses study by Birhanu Y et al. [24] focused on the pooled prevalence of SSI and its aetiology in Ethiopia. Also, the study had limitations, the papers included used only phenotypic laboratory methods and the result did not display AMR data. Thus, there have always been ambiguities in the interpretation of the findings when using phenotypic bacterial identification methods. In this study, to avoid the ambiguity in the interpretation of strain identification, we employed Matrix-Assisted Laser Desorption/Ionization Time-of-Flight Mass Spectrometry (MALDI TOF) technique for the confirmation of bacterial isolates. Furthermore, this is comprehensive study with a large sample size conducted to determine the bacterial profile of surgical site infection and antimicrobial resistance patterns at four major hospitals in Ethiopia.

## Methods

### Study site and design

**Fig. 1:**
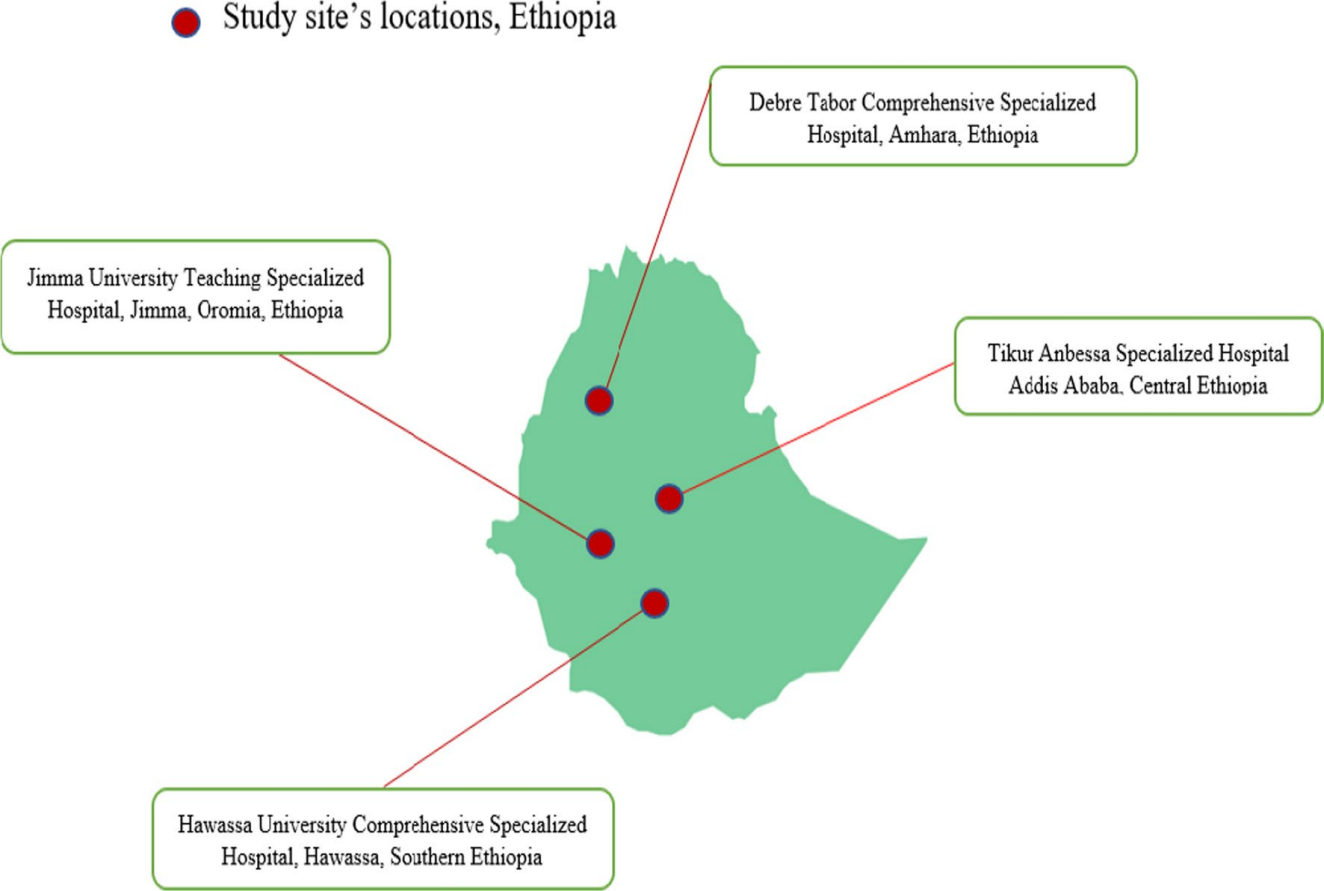
The map of the geographic locations of the four referral hospitals selected for this study

A multicentre cross-sectional study was conducted between July 2020 and August 2021 at four selected hospitals in Northern, Central, Southern and Southwest Ethiopia. The study was conducted in purposively selected University Teaching Hospitals in Ethiopia, namely, Debre Tabor Comprehensive Specialized Hospital (DTCSH), Hawassa University Teaching Hospital (HUTH), Jimma University Teaching Hospital (JUTH), and Tikur Anbessa Specialized Hospital (TASH) (Fig. 1).

*DTCSH* comprehensive specialized hospital provides health service to over 5 million people located in Debre Tabor town of South Gondar Administrative Zone, Amhara Regional State. Debre Tabor town is about 98 km away to the East of Bahir-Dar (the capital of Amhara regional state) and about 666 km North of Addis Ababa (the capital of Ethiopia). It is the only specialized hospital in the south Gondar Zone having over 400 beds.

The hospital provides surgery, pediatrics, emergency, maternity, gynecologic/obstetric, and psychiatric, including other departments. In addition, the hospital serves as a teaching centre for the region.

*HUCSH* is located in Hawassa city in Southern Ethiopia, 280 km from Addis Ababa. HUCSH is one of the largest health facilities in the Southern part of the country and provides teaching, public health services and research activities. It serves more than 20 million people locally and in the neighbouring regions. Currently, the hospital has over 400 beds and provides patient care to 90,200 outpatients, 18,100 hospitalized patients and 1100 emergency cases annually.

*TASH* is the teaching hospital of Addis Ababa University located in Addis Ababa, the capital of Ethiopia and the largest specialized hospital in Ethiopia, with over 700 beds. It is also an institution where specialized clinical services that are not available in other public or private institutions are rendered to the whole nation.

The TASH has 200 doctors, 379 nurses and 115 other health professionals dedicated to providing health care services. The various departments, faculties and residents under specialty training in the School of Medicine provide patient care in the hospital.

In their outpatient and inpatient units, the hospital offers a variety of services. They also have microbiology laboratories that perform culture and antimicrobial sensitivity testing.

While three hospitals had established microbiology laboratories the DTCSH had started performing bacteriological culture and antimicrobial susceptibility testing at the time of this study. Therefore, with the help of Armauer Hansen Research Institute (AHRI), DTCSH and my home institutions Debre Tabor University we established a bacteriology laboratory, which was used for wound culture processing and antimicrobial susceptibility testing.

### Patient recruitment and sample size calculation

The source population the study participants drawn were all patients with suspected cases of SSI who were admitted for elective and emergency surgery. Those who developed signs and symptoms of SSI within 30 or 90 (received implant) days and gave consent and/or assent to participate in the study were enrolled and decision to identify eligible patients as SSI cases were done by attending physicians. All age groups were included, but patients who had been on antibiotic treatment within the preceding ten days, SSI later than 30 days after the operation, or refused to give assent or consent (participate in the study) as well as patients with infected burn wounds, were excluded from the study. A total of 752 clinically diagnosed cases of SSI from different wards were enrolled in the study. The sample size was calculated based on a single proportion sample size estimation formula (n = Z^2^ P (1—P) /d^2^) using a proportion of 20% [25]. As this was a multicenter study, to increasing the sample size a precision (d) of 0.03 was used, where Z stands for Z statistic with a level of confidence of 95%, and the Z value of 1.96. With a 10% non-response rate, the total sample size came to 752. A convenient sampling technique was used to recruit study participants until the required sample size was achieved, and proportional allocation was made among different hospitals based on the patient flow across the four study sites.

### Operational definitions

*Surgical site infection* occurs near or at the incision site and/or deeper underlying tissue spaces and organs within 30 days of a surgical procedure performed (or up to 90 days for implanted prosthetics) [3].

*Clean wound* where no inflammation is encountered and the respiratory, alimentary or genitourinary tracts were not entered.

*Clean contaminated* wound is where the respiratory, alimentary or genitourinary tracts were entered but without significant spillage.

*Contaminated* when acute inflammation is encountered, or there is visible contamination of the wound.

*Dirty wound* wound in the presence of pus, where there is a previously perforated hollow viscous or compound/open injury more than four hours old [26].

*Antibiotic* a drug, which is products of fungi or bacteria that kills bacteria or inhibits their growth. Antibiotics are not effective against viruses (also referred to as an antimicrobial).

*Multidrug resistance (MDR)* refers to resistance at least one antimicrobial agent in three or more antimicrobial classes.

### Data collection

**Fig. 2:**
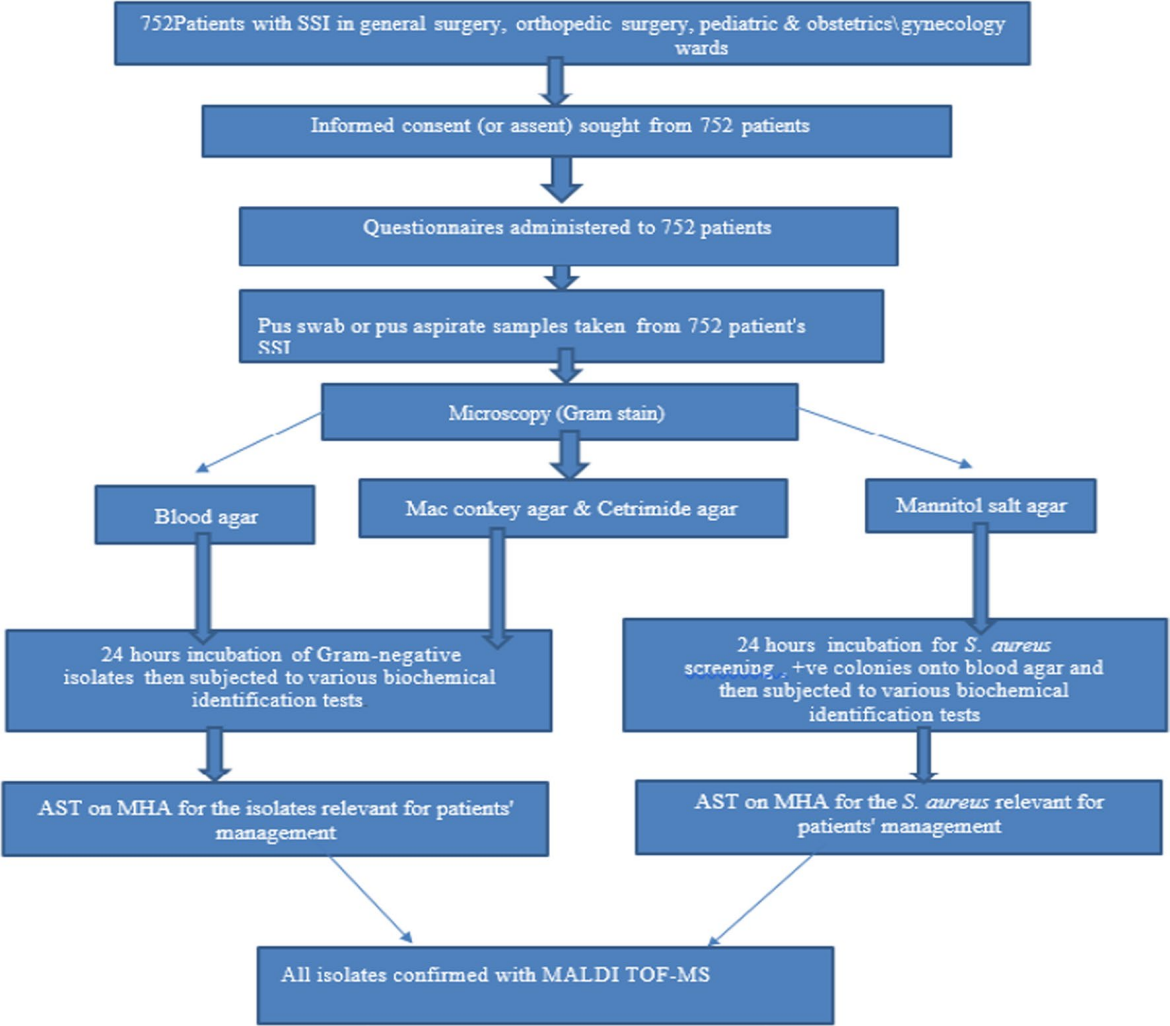
Laboratory workflow illustrating through patient recruitment to confirmation of the species of bacterial isolates

Professional nurses who had experience of wound swabs sample collection and microbiologists who were working in the bacteriology laboratory were recruited as data collectors. Training on socio-demographic and clinical data collection using structured questionnaires, wound swab sample collection and sample transportation to bacteriology laboratories and culture were given to all data collectors. Wound swab cultures, bacterial identification and drug susceptibility testing were performed in accordance with a standardized laboratory protocol that was uniformly applied in all study sites (Fig. 2). The findings of each culture were communicated to attending physicians for patient management. All bacterial strains were stored at − 80 °C and transported to the Armauer Hansen Research Institute (AHRI) and Sweden for further characterization. The isolates were transported to Sweden utilizing a triple packaging method with dry ice, specifically engineered for the safe carriage of Category A and B for Infectious and non-infectious substances, in accordance with UN regulation UN3373 for DNA samples and UN2814 for bacterial isolates.

### Sample collection and transportation

Trained personnel collected SSI discharge aseptically (pus, pus aspirates, and wound swabs) with sterile syringe with needle pus aspirate or sterile cotton-tipped swabs from inside to outside. After cleaning the infected area with 10% povidone-iodine, a sterile cotton-tipped swab was placed in the center, and the rolling technique was used. All wound swabs were dipped in modified Stuart's Transport Medium and immediately transported to the bacteriology laboratory for culture and drug susceptibility testing within 1 h. Following that, all collected specimens were processed for the identification of bacteria implicated in SSIs.

### Biochemical tests

For the final identification of the isolates, the following biochemical tests were performed using colonies from pure cultures. For Gram-negative rods, gas production, sugar fermentation, H2S production, indole production, citrate utilization, lysine decarboxylase production, urea hydrolysis, and motility tests were used. Gram-positive cocci were identified using the results of the Gram reaction, catalase, coagulase, bacitracin, and optochin tests [27].

### Bacterial strain confirmation using matrix-assisted laser desorption ionization-time of flight mass spectrometry (MALDI-TOF MS)

The isolates were transported to Sweden utilizing a triple packaging method with dry ice, specifically engineered for the safe carriage of Category A and B Infectious and non-infectious substances. All bacteria were re-identified and confirmed using MALDI-TOF MS at the Clinical Microbiology Department of Uppsala University Hospital Uppsala, Sweden. From fresh cultures, a single colony of bacteria was smeared onto a MALDI-TOF plate, and the sample was air-dried. Next, 1 µl of formic acid was added to each cell and air-dried, and then 1 µl of MALDI matrix solution was applied to the cells and air-dried before reading. MALDI-TOF identification was automatically scored by the system software between 1 and 3 points. All isolates with scores two and above were accepted, and all results below 1.7 and flagged red were rejected. Samples with scores 1.7–2 and flagged yellow were re-analysed.

### Antimicrobial susceptibility testing (AST)

The antibiotics susceptibility tests were performed on Muller-Hinton agar (Oxoid) by using the Kirby-Bauer disk diffusion technique. Using a sterile wire loop, 3–5 pure colonies were transferred to a tube containing 5 mL of sterile normal saline (0.85% NaCl) and gently mixed until a uniform suspension formed. Standard inoculum density was adjusted to 0.5 McFarland units. The excess broth suspension was removed by tapping against the tube wall. The bacterial suspension was swabbed on MHA surface by using sterile swab then a set of antibiotic discs placed with sterile forceps at least 24 mm apart from one another [28]. All antibiotics discs were OXOID products (Oxoid Ltd, UK), and susceptibility of Gram-negative isolates was tested against: ampicillin (10 µg), gentamicin (10 µg), amikacin (30 µg), ciprofloxacin (5 µg), chloramphenicol (30 µg), ceftazidime (30 µg), cefotaxime (30 µg), ceftriaxone (30 μg), cefuroxime (30 µg), cefepime (30 µg), tetracycline (30 µg), amoxycillin + Clavulanate (20/10 μg), Trimethoprim-sulfamethoxazole (1.25/23.75 µg), ampicillin-sulbactam (10/10 µg), aztreonam (30 µg), meropenem (10 µg), Imipenem (10 µg), ertapenem (30 µg). Gram-positive isolates were tested against penicillin (10units), ampicillin (10 µg), vancomycin (30 µg), erythromycin (15 µg), ciprofloxacin (5 µg), cefoxitin (30 µg), clindamycin (30 µg), erythromycin (15 µg), doxycycline (30 µg), chloramphenicol (30 µg), gentamicin (10 µg), and oxacillin (5 µg), tetracycline (30 µg), [28]. Following that, the plates were incubated at 37 °C for 18–24 h. Each zone of inhibition was measured to the nearest millimeter, and classified as sensitive, intermediate, or resistant using the standard technique [28]. MDR was a bacterium that was simultaneously resistant at least one drug in three or more categories.

### Quality control

All specimens were collected according to the standard operating procedure (SOP) [21]. A double data entry method was used to ensure the accuracy of the data. The performance of all prepared media was checked by inoculating control strains, *E. coli* (ATCC 25922) and *S. aureus (*ATCC 25923), for each new batch of agar plates [22]. In addition, the sterility of culture media was checked by incubating 5% of the prepared media at 37 °C for 24–48 h. In addition, reagents for Gram-stain and biochemical tests were checked against control strains of *S. aureus* and *E. coli.* The 0.5 McFarland standard was used to standardize the inoculum density of the bacterial suspension for the susceptibility test. Each MALDI-TOF run also included quality control strains using *E. coli* (ATCC 25922) and *S. aureus (*ATCC 25923).

### Data analysis

The data were checked for completeness, missing values, and coding of questionnaires entered into Research Electronic Data Capture (RED-Cap) and exported to STATA version 16.0. Frequencies and cross-tabulations were used to summarize descriptive statistics (median, percentages or frequency). Associations of possible risk factors with SSIs was assessed using bivariate and multivariate logistic regression to study the effect of independent variables on the dependent variables*.* P-value less than 0.05 were considered statistically significant.

### Ethical considerations

The Department of Medical Microbiology, Immunology, and Parasitology (DMIP) and the AHRI/ALERT Research Ethics Committee (AAREC) reviewed and approved the study, and institutional review board (IRB) approval was obtained from Addis Ababa University's College of Health Sciences and AAREC, AAUMF03-008/2020. Written permission letter was obtained from each study site before starting the data collection. The purpose and procedures of the study was explained to the study participants, participants’ parents or guardians before recruitment to the study. Those study participants who gave written informed consent and those children whose parents or guardians gave informed assent were selected and enrolled in this study. Results obtained from all patients were communicated to attending physicians and all patient’s information was kept confidentially.

## Results

In the present study, a total of 752 patients from four different hospitals were investigated for SSIs. The number of patients from DTCSH was172, and the numbers from HUCSH, JUSTH, and TASH, were184, 193, and 203, respectively (Table 1). Of the 752 study participants whose SSI discharge was inoculated onto growth media, 65.5% (493 /752**)** showed bacterial growth (Table 1). DTCSH had the highest percentage of positive cultures (78.5%), followed by JUTSH (65.3%), and HUCSH and TASH, respectively, had 65.2% and 55.7% of SSI bacterial growth (Table 1). The study participants age ranged from 3 days to 85 years with median of 28 years and 418 (55.6%) were males. Approximately 487 (64.8%) of patients had deep SSI, 454 (60.4%) preoperative hospital stay > 7 days, 619 (82.4%) history of hospital admission, 388 (52.9%) had previous use of antibiotics, 448 (59.6%) had smoking history, 506 (67.2%) of surgical procedures were emergency surgery, 724 (96.2%) of patients with clean or clean contaminated wounds dominated the wound class, 548 (72.8%) required antimicrobial prophylaxis before the procedure, and 55.3% underwent surgeries lasting greater than an hour (Table 1).

**Table 1:** Socio-demographic characteristics and clinical data

Characteristic	Frequency (%)
*Hospitals*	
DTCSH (n = 172)	172 (22.9)
Growth	135 (78.5)
No growth	37 (21.5)
HUCSH (n = 184)	184 (24.5)
Growth	120 (65.2)
No growth	64 (34.8)
JUSTH (n = 193)	193 (25.7)
Growth	126 (65.3)
No growth	67 (34.7)
TASH (n = 203)	203 (27)
Growth	113 (55.7)
No growth	90 (44.3)
Sex	
Male	418 (55.6)
Female	334 (44.4)
Age in (year)	
≤ 18	159 (21.1)
19–40	419 (55.7)
41–60	130 (17.3)
≥ 61	44 (5.9)
Surgical site infection	
Superficial	265 (35.2)
Deep	487 (64.8)
Preoperative hospital stay	
≤ 7	298 (39.6)
> 7	454 (60.4)
History of hospital admission	
Yes	133 (17.6)
No	619 (82.4)
Previous use of antibiotics	
Yes	388 (52.6)
No	364 (47.4)
Alcoholic	
Yes	67 (8.9)
No	685 (91.1)
Smoking	
Yes	444 (59)
No	308 (41)
Nature of surgery	
Elective	246 (32.7)
Emergency	506 (67.3)
Type of surgery	
Clean/clean contaminated surgery	724 (96.2)
Contaminated surgery	28 (3.8)
Timing of surgical antimicrobial prophylaxis	
Before the operation	548 (72.8)
During the operation	204 (27.2)
Duration of operation	
≤ 1 h	336 (44.7)
> 1 h	416 (55.3)

Bivariate and multivariable logistic regression analyses were used to see the relationship between the independent variables over the dependent variable. On bivariate regression analysis, male sex, age ≥ 61, SSI type, preoperative hospital stays, history of hospital admission, previous use of antibiotics, smoking, emergency surgery, and duration of operation ≥ 1 h had a statistically significant association with the occurrence of SSI. The type of surgery (wound), alcohol history and the timing of prophylactic antibiotics ≥ 1 h had no statistically significant association (Table 2). The result of the multivariate regression showed that ages ≥ 61 years (AOR = 2.83, 95% CI: 1.02–7.99; P 0.046), prolonged duration of hospital stay (AOR = 4.15, 95% CI: 2.87–6.01; P 0.000), history of previous antibiotics use (AOR = 2.83, 95% CI: 1.06–2.80; P 0.028), history of smoking (AOR = 2.35, 95% CI:1.44–3.83; P 0.001), emergency surgery (AOR = 3.24, 95% CI: 2.29–4.77; P 0.000), and duration of operation (AOR = 0.27, 95% CI: 0.181–0.39; P 0.000) were significant risk factors (Table 2).

**Table 2:** Bivariate and multivariate analysis to identify factors associated with patient demographic and clinical characteristics with surgical site infection culture showed growth

Characteristics	Bacterial growth n (%)		P-value	Crude-OR (95%CI)	Adjusted- OR (95%CI)	P-value
	Growth	No growth				
Sex						
Male	309 (41.1)	109 (14.5)	0.194	0.81 (0.6293– 1.112)	.94 (0.6420–1.373)	0.75
Female	233 (31)	101 (13.4)			1	
Age in (year)						
≤ 18	119 (15.8)	40 (5.3)	0.181	1.15 (0.937–1.421)	1.263 (0.7855–0.031)	0.33
19–40	287 (38.2)	132 (17.6)			1	
41–60	98 (13)	32 (4.3)			1.260 (0.7489–2.122)	0.38
≥ 61	38 (5.1)	6 (0.8)			2.83 (1.017–7.898)	**0.046**
SSI						
Superficial	183 (24.3)	82 (10.9)	0.149	1.271 (0.918–1.762)	1	
Deep	359 (47.7)	128 (17)			0.992 (0.6783–1.451)	0.97
Preoperative hospital stay						
≤ 7	145 (19.3)	124 (16.5)	0.000	3.908 (2.807–5.442)	1	
> 7	397 (52.8)	86 (11.4)			4.15 (2.87–6.01)	**0.000**
Previous use of antibiotics						
Yes	326 (43.3)	72 (9.6)			2.83 (1.06–2.80)	**0.028**
No	216 (28.7)	138 (18.4)	0.000	2.641 (1.897–3.673)	1	
Alcoholic history						
Yes	54 (7.2)	13 (1.7)	0.093	1.711 (0.915–3.201)		
No	488 (64.9)	197 (26.2)				
Smoking history						
Yes	359 (47.7)	89 (11.8)			2.35 (1.44–3.83)	**0.001**
No	186 (24.7)	118 (15.7)	0.000	2.646 (1.915–3.656)	1	
Nature of surgery						
Elective	147 (19.5)	99 (13.2)	0.000	2.395 (1.726–3.323)	1	
Emergency	395 (52.5)	111 (5.5)			3.24 (2.298–4.77)	**0.000**
Type of surgery						
Clean/Clean contaminated surgery	518 (68.9)	206 (27.4)	0.09	1.578 (0.925–2.691)		
Contaminated surgery	24 (3.2)	4 (0.5)				
Timing of surgical antimicrobial prophylaxis						
Before the operation	150 (19.9)	58 (7.7)	0.984	0.996 (0.698–1.421)		
During the operation	392 (52.1)	152 (20.2)				
Duration of operation						
≤ 1 h	198 (27.7)	138 (17)			1	
> 1 h	344 (45.7)	72 (9.6)		0.332 (0.239–0.461)	0.266 (0.181–0.392)	**0.000**

### Frequency and distribution of identified bacterial isolates

**Fig. 3:**
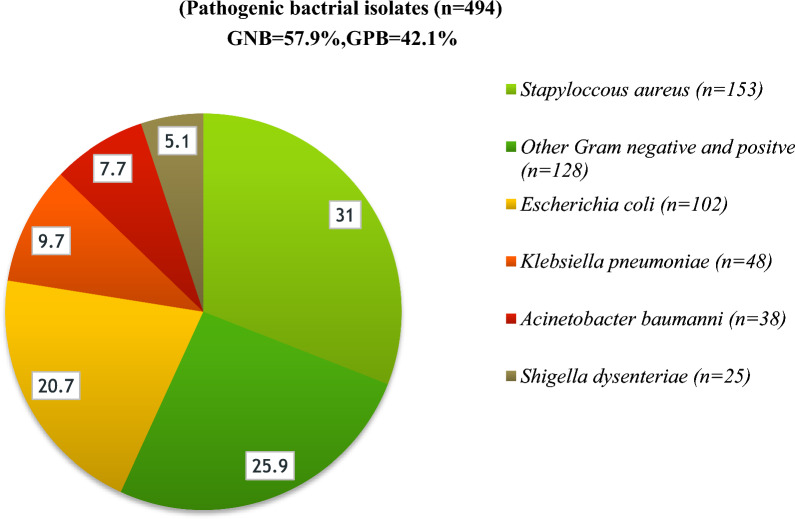
Frequency and distribution of bacteria isolated from patients investigated for surgical site infection at four different hospitals in Ethiopia. *GNB* Gram-negative bacteria, *GPB* Gram-positive bacteria, Other GN and GP (n = 128): *Raoultella ornithinolytica (n* = *1), Stenotrophomonas maltophilia (n* = *1), Acinetobacter soli (n* = *2), Acinetobacter pitti (n* = *2), Acinetobacter lactucae (n* = *1), Pseudomonas plecoglossicida (n* = *1), Pantoea ecurina (n* = *1), Citrobacter freundii (n* = *1), Citrobacter sedlakii (n* = *2), Providencia rettgeri (n* = *2), Alcalignes faecalis (n* = *2), Proteus mirabilis (n* = *4), Morganella morganii (n* = *1), Aerococcus viridans (n* = *3), Bacillus flexus(n* = *1) Paenibacillus tylopili (n* = *1), Enterobacter cloacae (n* = *19), Enterobacter asburiae (n* = *1), Enterobacter bugandensis (n* = *4), Enterobacter hormaeche (n* = *5), Enterococcus faecium (n* = *8): Enterococcus gallinarum (n* = *3), Enterococcus hirae (n* = *2), Enterococcus durans (n* = *2), Staphylococcus hominis (n* = *4), Staphylococcus haemolyticus (n* = *3), Staphylococcus warneri (n* = *2), Staphylococcus sciuri (n* = *6), S. epidermidis (n* = *8)*

**Fig. 4:**
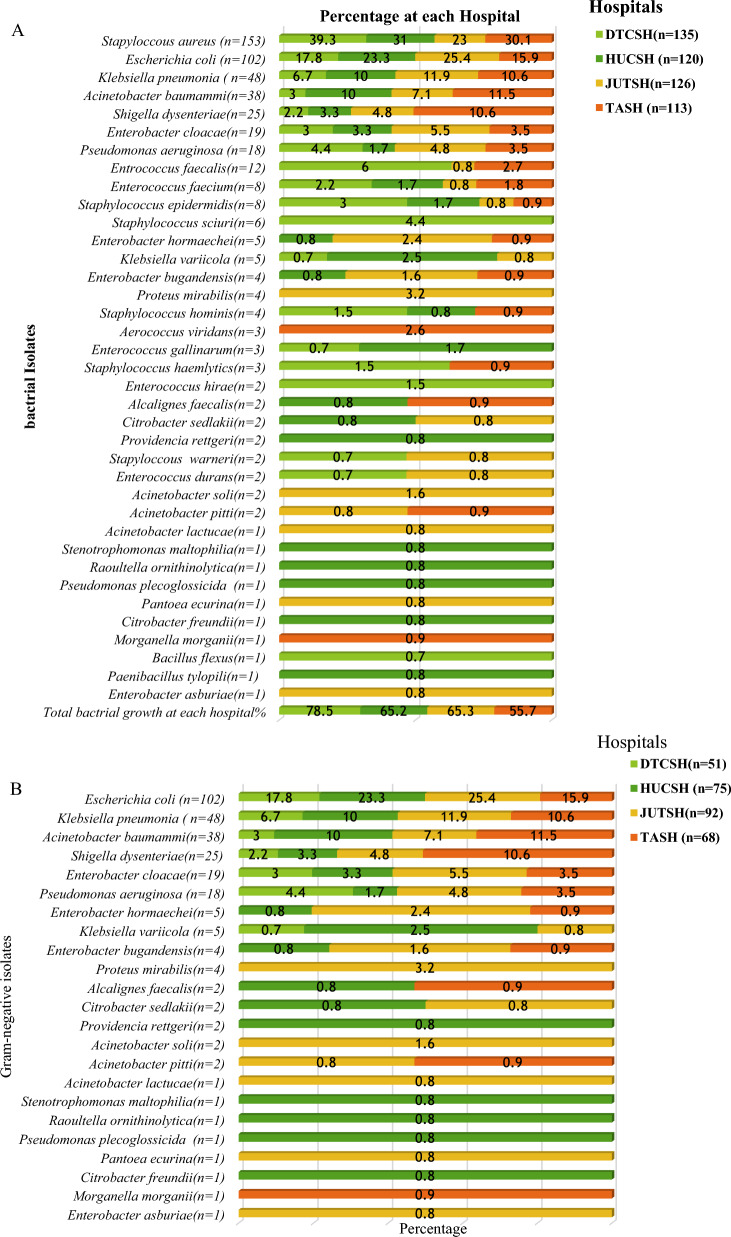

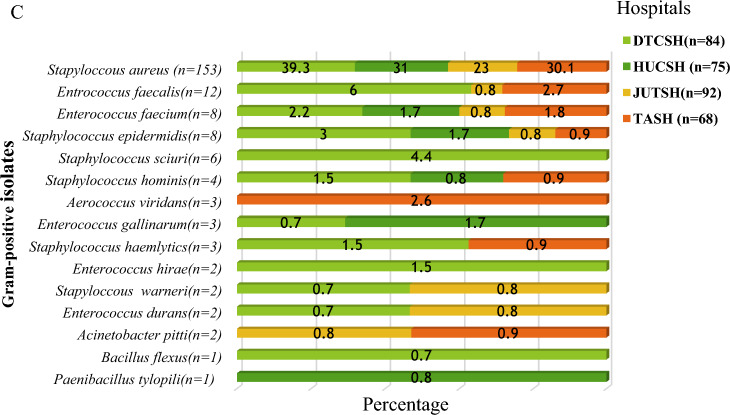
Frequency and distribution of bacterial isolates from the total number of bacteria isolated at each hospital **A** total identified bacteria at each site, **B** Gram-negative isolates and **C** Gram-positive isolates. *DTCSH* Debre Tabor Comprehensive Specialized Hospital, *HUCSH* Hawassa University Comprehensive Specialized Hospital, *JUTSH* Jimma University Teaching Specialized Hospital, *TASH* Tikur Anbessa Specialized Hospital, *n* number of bacterial isolates

The total number of pathogenic bacterial isolates were 65.7% (494/752**)** from all SSI culture (Figs. 3, 4A). Gram-negative were 57.9% (286/752**)**, and Gram positive 42.1% (208/752) according to Fig. 2B, C). Of these, 2.6% (13/493**)** of cultures were a mixture of two colony types, while 2.4% (12/493**)** wer**e** commensals or contaminants and 97.4% showed single bacterial growth. Species of the mixed cultures were *Raoultella ornithinolytica, Paenibacillus tylopili*, *S. aureus* and coagulase negative *staphylococci.* Among the identified types of bacteria, *Staphylococcus aureus* was the predominant one (31%), followed by *E. coli* (20.7%) and *Klebsiella pneumonia* (9.8%) among SSIs (Fig. 1). Other less frequently detected species were *Acinetobacter baumannii* (7.6%)*, Enterobacter cloacae* (5.1%)*, Pseudomonas aeruginosa* (3.7%)*, Klebsiella variicola, and Enterobacter hormaeche* (1% each). Diverse species of *Acinetobacter, Enterobacter, Enterococcus, Staphylococcus, Aerococcus, Bacillus, Citrobacter,* and *Pseudomonas* were identified. While Gram-positives was found at all four hospitals (42.1%), it was mainly detected at DTCSH (40.4%), with 21.6%, 21.6%, and 16.3% isolated at TASH, HUCSH, and JUTSH, respectively (Fig. 2B). In addition, *Raoultella ornithinolytica, Stenotrophomonas maltophilia, Pantoea ecurina, Providencia rettgeri, Alcalignes faecalis, and Morganella morganii* were detected as rare bacterial pathogens. Figure 2A shows the frequency and distribution of Gram-negative bacterial isolates at the four hospitals.

### Antibiotic resistance pattern of SSI bacterial isolates

The predominant isolate from Gram-positives, *S. aureus*, revealed a high level of resistance toward penicillin 90.1%, and ampicillin 76.5%, while 7.8%, 10.6%, and 12.4% of the isolates were resistant to clindamycin, chloramphenicol, and gentamicin respectively but 100% of *S. aureus* were sensitive to vancomycin (Table 3). All isolates of *S. aureus* showed multiple drug resistance (resistance to two or more drugs).

**Table 3:** Antimicrobial resistance pattern of Gram-positive bacteria isolated from patients diagnosed with surgical site infection in Ethiopia: a multicenter prospective cross-sectional study 2022

Gram-positive isolates	(%) of resistance to antimicrobial agents													
	P	AMP	E	TE	FOX	OXA	DOX	SXT	CPR	CN	CHL	DC	V	MDR%
*S. aureus (n* = *153)*	90.2	76.5	43.7	43	20	20	23.8	24.2	20	12.4	10.6	7.8	0	100
*Other staphylococcus spp.*														
*S. epidermidis (n* = *8)*	75	75	37.5	62.5	12.5	12.5	25	25	25	25	12.5	12.5	0	73.9
*Staphylococcus sciuri (n* = *6)*	100	100	50	50	100	100	50	50	33	33	33	25	0	
*Staphylococcus hominins (n* = *4)*	75	75	75	50	25	25	50	50	0	75	25	25	0	
*Staphylococcus haemolyticus (n* = *3)*	100	67	67	67	33	33	67	33	0	67	67	67	33	
*Staphylococcus warneri (n* = *2)*	100	100	100	50	50	50	50	50	50	50	50	NA	0	
Total (n = 176)	88.6	77.3	45.5	44.9	22.7	22.7	26.7	24.4	20.5	16.5	17.6	9.7	0	96.6
*Entrococcus faecalis (n* = *12)*	NA	66.7	66.7	75	NA	NA	NA	NA	NA	NA	66.7	NA	NA	
*Enterococcus faecium (n* = *8):*	NA	75	100	75	NA	NA	NA	NA	NA	NA	75	NA	NA	
*Enterococcus gallinarum* (n = 3)	NA	100	NA	67	NA	NA	NA	33	NA	NA	67	NA	NA	
*Enterococcus hirae (n* = *2)*	NA	50	50	50	NA	NA	NA	NA	NA	NA	50	NA	NA	
*Enterococcus durans* (n = 2)	NA	100	50	50	NA	NA	NA	NA	NA	NA	50	NA	NA	
Total (n = 27)	NA	70.4	66.7	59.3	NA	NA	NA	NA	NA	NA	66.7	NA	NA	
*Other gram positives*														
*Aerococcus viridans* (n = 3)	100	100	67	33	NA	NA	NA	67	100	67	67	NA	NA	
*Bacillus flexus* (n = 1)	NA	100	100	100	NA	NA	NA	0	100	0	0	NA	NA	
*Paenibacillus tylopili* (n = 1)	NA	100	100	100	NA	NA	NA	100	100	0	0	NA	NA	
Total (n = 5)	20	60	80	60	NA	NA	NA	80	100	40	40	NA	NA	

*P* Pencillin, *AMP* Ampicillin, *E* Erytromycin, *TE *Tetracycline, *FOX*: Cefoxitin, *OXA* Oxacillin, *DOX* Doxycycline, *SXT* Trimethoprim-Sulfamethoxazole, *CPR* Ciprofloxacin, *CN* Gentamicin, *CHL* Chloramphenicol, *DC* Clindamycin, *V* Vancomycin, *NA* not applicable, *MDR* Multidrug resistance

Cefoxitin, which is a surrogate marker of methicillin, showed 22.7% resistance against *S. aureus*. *Enterococcus* species showed 70.4% resistance to ampicillin and 66.7% to erythromycin. Table 3 shows the AMR pattern of Gram-positive bacteria.

The *Enterobacteriaceae* showed high resistance toward ampicillin (93.2%), ceftriaxone (90.5%), cefuroxime (88.7%), aztreonam (82.9%), ceftazidime (80.6%), cefepime (77%), ampicillin-sulbactam (76.1%), trimethoprim-sulfamethoxazole (77.5%), tetracycline (72.5%), amoxicillin-clavulanic acid (71.2%) (Table 4).

**Table 4:** Antibiotics resistance patterns of Gram-negative bacteria isolates among patients with surgical site infection in Ethiopia: a multicenter prospective cross-sectional study 2022

Gram-negative isolates		Resistance to antimicrobial agents (%)																	
	AMP	AMC	CHL	CRO	SXT	CN	AK	CXM	CTX	CPR	CAZ	FEP	IMP	MEM	ET	TE	SAM	ATM	MDR%
*E. coli (n* = *102)*	94.1	68.6	41.2	99	71.5	57.8	10.8	73.5	89.2	73.5	79.4	76.5	11.8	9.8	24.5	70.6	72	64	96.1
*K. pneumonia (n* = *48)*	100	91.7	45.8	93.8	64.6	70.8	33.3	77.1	93.8	62.5	85.5	81.25	29.2	41.7	43.8	66.7	85.2	96.8	95..9
*E. cloacae complex (n* = *29)*	96.6	79.3	62.1	89.7	75.7	58.6	24.1	72.4	86.2	55.2	79.3	79.3	37.9	17.2	31	62.1	71.4	63.1	79.3
*S. disentriae (n* = *25)*	100	28	32	92	64	28	16		84	44	76	76	20	20	20	48	66.9	66.5	82
*K. variicola (n* = *5)*	100	40	40	100	60	60	0	60	0	40	100	80	40	0	20	60	71.9	62.2	100
*P. mirabilis (n* = *4)*	100	25	25	75	50	50	0	75	25	50	50	75	0	0	0	50	66.1	65.2	100
*Rare Enterobacteriaceae isolates (n* = *9)*	88.8	88.8	44.4	77.8	44.4	66.7	22.2	77.8	77.8	66.7	77.8	66.7	33.3	11.1	22.2	55.5	45.1	38.7	100
*Total Enterobacteriaceae isolates (n* = *222)*	93.2	71.2	62.2	90.5	77.5	60.1	24.3	88.7	90.5	81.1	80.6	77	20.3	17.6	32.9	72.5	76.1	82.9	93.3
*Acinetobacter species*																			
*A. baumammi (n* = *38)*	–	–	–	94.7	81.4	86.8	42.1	–	95.3	73.7	89.5	84.2	65.9	84.2	92.1	–	63.1	–	95
*Other Acinetobacter species (n* = *5)*	–	–	–	95.3	20	60	0	–	95.3	40	60	60	20	40	100	–	40	–	60
*Total Acinetobacter species (n* = *43)*		–	–	95.3	86	13.5	39.5	–	95.3	69.7	81.4	86	67.4	14.9	90.7	–	67.4	–	93
*Pseudomonas species*																			
*P. aeruginosa (n* = *18)*	–	–	–	–	–	47.8	27.8	–	–	22.2	66.7	55.5	5.5	0	38.9	–	–	66.7	77.8
*P. plecoglossicida (n* = *1)*	–	–	–	–	–	0	0	–	–	100	100	100	0	0	100	–	–	100	100
*Total pseudomonas species (n* = *19)*	–	–	–	–	–	47.3	21.1	–	–	26.3	68.4	57.9	5.3	0	42.1	–	–	68.4	73.8

*AMP* Ampicillin, *AK* Amikacin, *AC* Amoxicillin-Clavulanic Acid, *ATM* Aztreonam, *CHL* Chloramphenicol, *CN* Gentamicin, *CRO* Ceftriaxone, *FEP* Cefepime *SXT* Trimethoprim-Sulfamethoxazole, *CPR* Ciprofloxacin, *CXM* Cefuroxime, *CTX* Cefotaxime, *ET* Ertapenem, *IPM* Imipenem, *MEM* Meropenem, *TE* Tetracycline

Low resistance frequency of *Enterobacteriaceae* was detected for amikacin (24.3%), imipenem (20.3%), meropenem (17.6%), and ertapenem (32.9%) (Table 4).

**Fig. 5:**
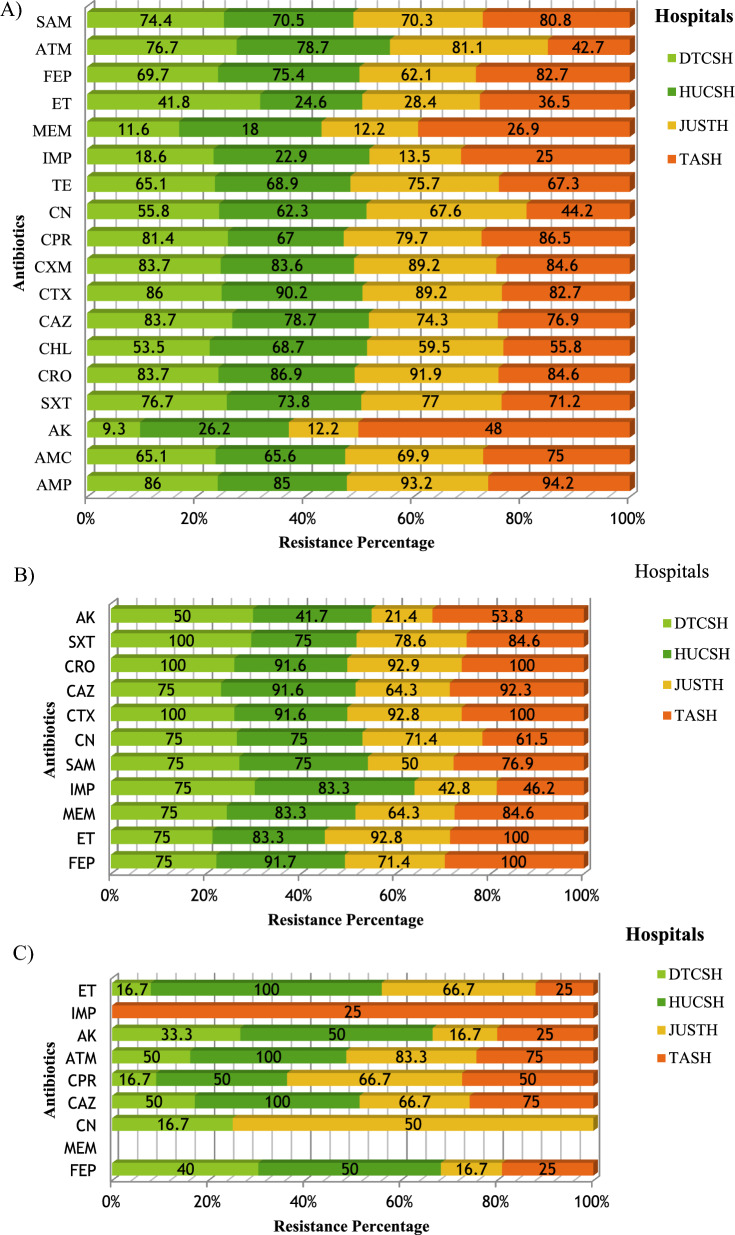
Frequency of antibiotic resistance at four hospitals; **A** *Enterobacteriaceae* **B** Acinetobacter species **C** Pseudomonas species. The percentage represents the numbers of resistant isolates, out of the total number of isolates at all hospitals. *AMP* ampicillin, *AMC* amoxicillin/clavulanate, *AK* amikacin, *SXT* trimethoprim-sulfamethoxazole, *C* chloramphenicol, *CAZ* ceftazidime, *CTX* cefotaxime, *CRO* ceftriaxone, *CXM* cefuroxime, *CIP* ciprofloxacin, *CN* gentamicin, *TE* tetracycline, *ATM* aztreonam, *SAM* ampicillin-sulbactam, *FEP* cefepime, *IMP* Impemene, *MEM* meropeneme, *ET* Ertapeneme, *DTCSH* Debre Tabor Comprehensive Specialized Hospital, *HUCSH* Hawassa University Comprehensive Specialized Hospital, *JUTSH* Jimma University Teaching Specialized Hospital, *TASH* Tikur Anbessa Specialized Hospital

The resistance of *Enterobacteriaceae* to meropenem and imipenem was (11.6%,18.6%), (18%, 22.9%), (12.2%,13.5%) and (26.9%,25%) at DTCSH, HUCSH, JUTSH, and TASH, respectively (Fig. 5A).

The predominant isolate, *E. coli* (n = 102) revealed a high level of resistance to ampicillin (94.6%), ceftriaxone (99%), cefotaxime (93.8), ceftazidime (79.4%), cefepime (77%), cefuroxime (73.5%), ampicillin-sulbactam (72%), trimethoprim-sulfamethoxazole (71.5%), tetracycline (70.6%), and low-level resistance to gentamicin (57.8%), chloramphenicol (41.2%), ertapenem (24.5), imipenem (11.6%), amikacin (10.8%), meropenem (9.8).

*K. pneumoniae* (n = 48) were resistant to ampicillin (100%), ceftriaxone (100%), cefotaxime (93.8%), amoxicillin-clavulanic acid (91.7%), ceftazidime (88.5%), cefepime (81.2%), cefuroxime (77.1%), tetracycline (66.7%), ertapenem (43.8%), meropenem (41.7%), amikacin (33.3%), imipenem (29.2%). Amikacin and meropenem were 100% effective against all of the isolates of *Klebsiella variicola and Proteus mirabilis.* In the non-fermenter group, *A. baumannii* showed the highest resistance to cefotaxime (95.3%), ertapenem (92.1%), ceftazidime (89.5%), gentamicin (86.8%), cefepime (84.2%), meropenem (84.2%), and SXT (81.4%). In addition, *A. baumannii* has lower-level resistance to imipenem (65.9%) and ampicillin-sulbactam (63.1%) (Table 4). The resistance frequency of Acinetobacter species to meropenem at DRH, HUCSH, JUSTH, and TASH was 75%, 83.3%, 42.8%, and 46.2%, respectively (Fig. 3B). *P. aeruginosa* showed minimal resistance to ceftazidime (66.7%), cefepime (55.5%), gentamicin (47.8%), ciprofloxacin (22.2%), and amikacin (10.5%) (Table 4). In addition, 100% and 94.5% of Pseudomonas species were sensitive to meropenem and imipenem, respectively (Table 4, Fig. 5C).

### Multidrug resistance

**Fig. 6:**
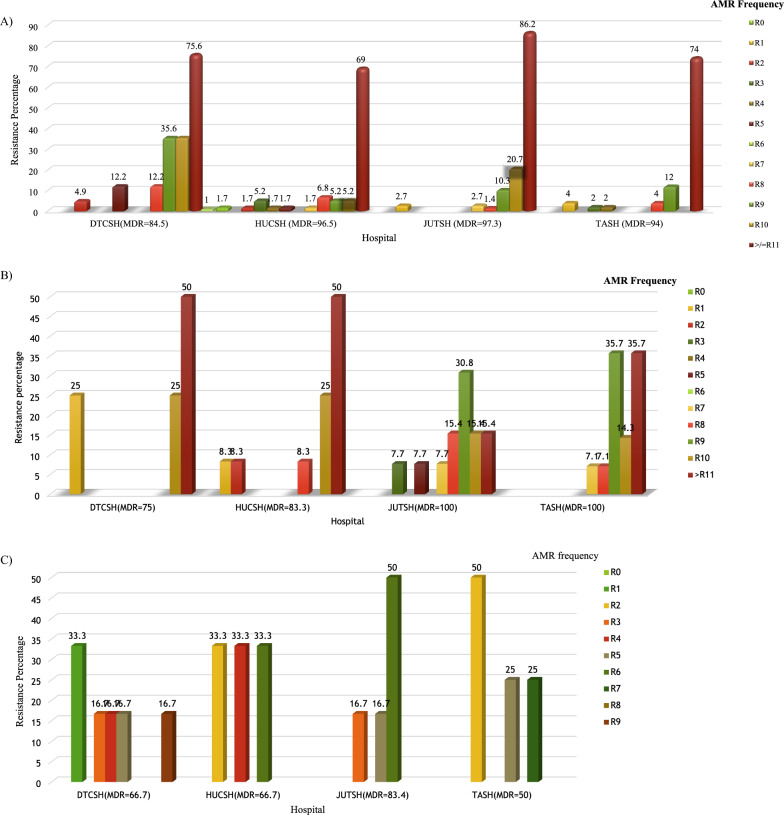
Frequency of multidrug resistance at four hospitals. A *Enterobacteriaceae* B Acinetobacter species C Pseudomonas species. Percentages represent the number of resistant isolates out of the total number of isolates at each hospital. Debre Tabor Comprehensive Specialized Hospital (DTCSH), *HUCSH* Hawassa University Comprehensive Specialized Hospital, and Jimma University Teaching Specialized Hospital (JUTH) and Tikur Anbessa Specialized Hospital (TASH), *MDR* multidrug resistance

The overall Multidrug resistance (MDR) to three or more antibiotics was observed in 100% of *S. aureus* (Table 3) and 93.3% *Enterobacteriaceae* (Table 4). *Enterobacteriaceae* that showed MDR to eight (R-9), nine (R-10) and ten (R-** ≥ **11) antibiotics from different groups had a frequency of 6.3%, 6.7%, and 64.9%, respectively. Only 0.5% *Enterobacteriaceae* showed zero resistance (R-0) to all antibiotic classes tested, whereas 3.1% *Enterobacteriaceae* showed resistance to one antibiotic (R-1) class. For *Enterobacteriaceae*, the MDR frequency at DTCSH, HUCSH, JUSTH, and TASH was 84.5%, 96.5%, 97.3%, and 94%, respectively (Fig. 6A). *E. coli, K. pneumoniae, E. cloacae, S. dysenteriae, K. variicola, and P. mirabilis* showed an overall MDR frequency of 96.1%, 95.9%, 79.3%, 82%, 100%, and 100%, respectively. The overall MDR frequency of *A. baumannii* and *P. aeruginosa* was 95% and 77.8%, respectively. The MDR frequency for Acinetobacter species was 73% at DTCSH, 83.3% at HUCSH, 100% at JUTSH, and 100% at TASH (Fig. 4B). On the other hand, MDR frequency for Pseudomonas species was 66.7%, 66.7%, 83.3% and 50% at DTCSH, HUCSH, and JUSTH and TASH, respectively (Fig. 6C).

## Discussion

In surgically treated patients, post-operative SSI is still one of the leading causes of morbidity and mortality, and it increases the cost of health care due to repeated readmission. In order to effectively manage bacterial infection, it is crucial to identify the bacterial pathogens and choose an antibiotic that is efficient against the organism [1, 2].

As far as risk factors associated with the occurrence of SSI are concerned, in the present study, the likelihood of SSI occurrences among patients aged ≥ 61 years increased by a factor of 2.8. Similar findings have been conducted in Ethiopia [15, 16, 29] and elsewhere [30]. This might be due to a weakened immune response to infectious agents and poor nutritional status [31].

Patients who had a longer duration of hospital stay developed SSIs 4.1 times more frequently (P = 0.000) than those who had shorter time. This finding was in agreement with many studies in Ethiopia [29, 32, 33] and elsewhere [30]. This is a notable finding because it is associated with additional costs in a country with a staggering economy and healthcare system [34].

Similarly, the present study demonstrated that with previous use of antibiotics, patients had a 2.8 times higher chance of developing SSI than with non-previous use of antibiotics [35]. This could be because broad-spectrum antibiotics have a high risk of causing superinfection of resistant strains due to selective pressure [18, 19].

The type of surgery was also statistically associated with SSI in the present study. Undergoing emergency surgery showed approximately 3.24 times higher chances of acquiring SSIs when compared to elective surgery (P = 0.000), which complies with related studies [35].

The risk of developing SSI with smoking histories was found to be 2.35 times more than in those who did not have smoking history. There was a significant association between smoking patients’ history and SSI (P = 0.001). This finding was in agreement with smoking history were independent predictors of SSIs in multivariate logistic regression analysis [36]. Smoking weakens immunity and increases the risk of SSI [37].

The overall culture positivity rate from patients with SSI in the current study was 65.5%. Similar results reported from India (68%) [38]. Which was slightly smaller than results previously reported from Jimma (71.7%) [9]. In contrast, the current study was lower than reports from Tikur Anbessa (75.6%) [39], Gondar (83.9%) [40], and elsewhere (82%) [41]. Lower rates of positive culture were reported from India Bangladesh (61.8%) [42].

In this study, the Gram-negative bacterial isolation rate was greater (57.9%) than the Gram-positive isolates (42.1%), which is comparable with the study done in Addis Ababa [43]. On the other hand, the current study was lower in Gram- negative isolates than reports from Mizan-Tepi (73.2%) and higher in Gram-positive isolates (24.8%) [22]. This could be due to a difference in the study population.

In the current study, the profiles of bacterial isolates highly associated with SSI were *S. aureus* (31%), followed by *E. coli* (20.7%), and *K. pneumoniae* (8.9%). Studies from Gondar [44], Addis Ababa [45], and India [40] reported similar results. The high prevalence of *S. aureus* infection could be due to an endogenous source as well as environmental contamination.

In contrast to previous reports, a study from Addis Ababa found *E. coli* 23.1%, followed by multidrug-resistant Acinetobacter species 22.1% [34]. Similarly, in a study done by Jimma, *E. coli* was frequently identified and followed by Klebsiella spp. [41]. This variation in the distribution pattern of bacterial isolates may be due to the diversity of the study population, setting, and local antimicrobial usage pattern, which leads to the introduction of pathogens that may be resistant to currently used antibiotics.

Rare surgical site infections isolate including *Raoultella ornithinolytica*, *Stenotrophomonas maltophilia, Alcalignes faecalis, and Paenibacillus tylopili,* were identified. Such newly emerging bacteria-causing infections in SSI patients may result in future challenges [46–48]. These species, along with *K. variicola* and *Pantoea ecurina*, have never before been identified in patients in Ethiopia who were being evaluated for SSI. The isolation of these new SSI etiologies emphasizes the necessity of institution-based diagnostic and intervention practices.

In our finding, *S. aureus* revealed a high level of resistance to penicillin (88.6%) and ampicillin (77.3%), which was comparable to study in Turkey [46]. On the other hand, all isolated *S. aureus* were susceptible to vancomycin, and our finding was the same as earlier studies in Jimma [36] and Turkey [46].

Gram-negative bacteria showed higher resistance to ampicillin (93.2%), ceftriaxone (90.5%), cefuroxime (88.7%), aztreonam (82.9%), ceftazidime (80.6%), cefepime (77%), ampicillin-sulbactam (76.1%), trimethoprim-sulfamethoxazole (77.5%), tetracycline (72.5%), and amoxicillin-clavulanic acid (71.2%). Since β-lactam antibiotics are the most widely used antibiotics, many studies in Ethiopia [11, 34] and around the world have found similar resistant patterns [40]. This shows antibiotics require a periodic evaluation and the establishment of antibiotic policies for prophylaxis and treatment guidelines in the Ethiopian setting.

In this study, multidrug resistance (MDR) was observed (100%) in *S. aureus* and (93.4%) in Gram-negative bacteria, similar to findings from Bahir Dar [18].

In the current study, *A. baumannii* showed the highest to resistance cefotaxime (95.3%), ceftazidime (89.5%), gentamicin (86.8%), cefepime (84.2%), and trimethoprim-sulfamethoxazole (81.4%). Many studies have found that these organisms have a high resistance to the most commonly used antibiotics [34, 47, 48]. In addition, *A. baumannii* also showed remarkably high resistance to ertapenem (92.1%), meropenem (84.2%), and imipenem (65.9%).

Amikacin and meropenem were 100% effective against all of the isolates of* P. mirabilis* and *K. variicola* in our study. However, studies done in Mekelle [37], and elsewhere [29] showed that ciprofloxacin were effective against Proteus and Pseudomonas isolates. The differences maybe the rational use of antibiotics and the fact that the cost of the drugs may be higher relative to others, so people do not take these drugs for self-medication in the study area [49].

Carbapenem resistance among *Enterobacteriaceae* was 17.6%, 20.3%, and 32.9% to meropenem, imipenem, and ertapenem, respectively. Effective treatment options for *Enterobacteriaceae* were limited to amikacin, meropenem, and imipenem. The most frequent isolate *is E. coli, which* showed the highest resistance to ampicillin (ceftriaxone, cefotaxime, ceftazidime, cefepime, cefuroxime, ampicillin-sulbactam, trimethoprim-sulfamethoxazole, and tetracycline. Similar studies were conducted in Ethiopia [33] and Iraq [50]. This might be due to the indiscriminate use of antibiotics in both hospitals [18, 19].

In our study, an alarming level of carbapenem-resistant *E. coli* to ertapenem (24.5%), imipenem (11.8%)*,* and meropenem (9.8%) was detected; however, it was lower than in another study [51]. The cause of the higher rates compared to other settings may be irrational use or misuses of antibiotics. The discrepancy of antibiotics resistance across sites includes differences in the rational use of antibiotics [52].

### Strengths and limitation

The strength of this study was multicentred, enrollment of all age groups, a reasonably large sample size and re-characterizing bacteria using MALDI TOF–MS an advanced bacterial identification method, and the limitation were unable to investigate anaerobic bacterial and fungal agents due to limited laboratory resources at the hospitals.

## Conclusions

This multicenter study identified frequent and diverse Gram-negative and Gram-positive SSI etiologies without significant variation in primary etiologies between hospitals. Isolation of various newly emerging bacterial strains in all sites showed the growing epidemiology and diversity of SSI etiologies. *E. coli* and *S. aureus* were the leading Gram-negative and Gram-positive isolates, respectively. High antimicrobial resistance was detected with varying frequency between hospitals. Gram-positive isolates revealed maximum sensitivity to vancomycin and clindamycin, whereas, among Gram-negative isolates, amikacin, imipenem, and meropenem were the most effective antibiotics Furthermore, the overall ceftriaxone resistance is about 90.5%. Among the study participants, 72.1% took prophylaxis and developed SSIs. The finding of high levels of carbapenem resistance, especially towards ertapenem, is alarming.

Hence, to prevent the emergence and spread of MDR SSI, we recommend effective antimicrobial stewardship and antibiotic treatment to based on AST of the pathogens. At the national level, regular surveillance and monitoring of antimicrobial resistance patterns are indispensable. This includes the careful monitoring of the antibiotics used as prophylaxis and empiric treatment by the concerned authorities.

## Data Availability

The data sets generated during and/or analysed during the current study are available from the corresponding authors on reasonable request.

## Abbreviations

AAERC: AHRI/ALERT Ethics Review Committee
AHRI: Armauer Hansen Research Institute
AMR: Antimicrobial resistance
AST: Antimicrobial susceptibility testing
ATCC: American Type of Culture Collection
CDC: Center for Disease Control and Prevention
CLSI: Clinical Laboratory Standards Institute
DMIP: Department of Microbiology Immunology and Parasitology
DTTRH: Debre Tabor Teaching, and Referral Hospital
HUTH: Hawassa University Teaching Hospital
JUTH: Jimma University Teaching Hospital
MALDI-TOF MS: Matrix-assisted laser desorption ionization-time of flight mass spectrometry
MDR: Multi drug resistance
MHA: Mueller Hinton Agar
SOP: Standard operation procedure
SSI: Surgical site infections
STATA: South Texas Art Therapy Association
TASH: Tikur Anbessa Specialized Hospital
ESBL: Extended-spectrum beta lactamases
